# Correspondence and Concordance of Retrospective and Concurrent Responses to Physiotherapists and Sport Psychology Questionnaire Items

**DOI:** 10.3390/ijerph19095106

**Published:** 2022-04-22

**Authors:** Ashlee E. Groover, Britton W. Brewer, Daniel M. Smith, Judy L. Van Raalte, Christine N. May

**Affiliations:** 1 Department of Psychology, Springfield College, 263 Alden Street, Springfield, MA 01109, USA; agroover@springfieldcollege.edu (A.E.G.); jvanraal@springfieldcollege.edu (J.L.V.R.); christinemay811@gmail.com (C.N.M.); 2 Department of Physical Education and Health Education, Springfield College, 263 Alden Street, Springfield, MA 01109, USA; dsmith19@springfieldcollege.edu; 3 College of Health Sciences, Wuhan Sports University, 461 Luoyu Road, Wuhan 430079, China

**Keywords:** measurement, physical therapy, PSPQ, orthopedic, psychology, rehabilitation, validity

## Abstract

Orthopedic and sport-related injuries are a major public health concern and a common reason for referral to physical therapy. The use of psychological techniques by physical therapists has been assessed in research studies primarily with retrospective self-report questionnaires that have not been validated against concurrent assessments of the same behaviors. The primary purpose of this study was to examine the extent to which the results obtained from physical therapists’ retrospective self-reports of their use of psychological techniques reflect their use of the techniques assessed concurrently. Physical therapists (*N* = 14) completed the Physiotherapists and Sport Psychology Questionnaire (PSPQ) at the beginning of this study and a checklist based on the PSPQ at the end of the sessions with patients (*N* = 306). Patients also completed the checklist at the end of the sessions. Across 12 psychological techniques, the physical therapists’ retrospective (PSPQ) responses showed relatively weak correspondence (mean *r* = 0.31) and poor concordance with their concurrent (checklist) responses. Compared to the physical therapists’ checklist responses, the patients’ checklist responses showed weaker correspondence (mean *r* = 0.03) and better concordance with the physical therapists’ PSPQ responses. The findings suggest that retrospective self-reports may not accurately reflect the use of psychological techniques by physical therapists and, consequently, that physical therapists should consider documenting their use of psychological techniques as close to their implementation as possible. Suggestions for improved assessment are provided.

## 1. Introduction

Orthopedic and sport-related injuries [1,2,3,4] are ubiquitous, have extensive adverse economic, physical, psychological, and social effects [3,4,5,6], and are a common reason for referral to physical therapy [7]. Within orthopedic and sports physical therapy, psychological factors are relevant to a wide range of issues of central importance, including adherence to rehabilitation, injury occurrence, pain, recovery of function, return to activity, and the working alliance between patients and practitioners [8,9]. Given their extensive interpersonal contact with patients, physical therapists are in an optimal position to implement basic psychological interventions to facilitate rehabilitation outcomes. Recognizing the potential role of psychology in physical therapy, investigators have examined the extent to which physical therapists use various psychological techniques in their practice over the past two decades [10,11]. In general, physical therapists in musculoskeletal rehabilitation settings have reported that they use goal setting and other motivational techniques (e.g., positive reinforcement, motivational interviewing, and effective communication) frequently and techniques such as mental imagery, relaxation, reducing depression, and teaching emotional control strategies infrequently [11].

In examining the use of psychological techniques by physical therapists, researchers have relied primarily on methods involving retrospective self-report (i.e., surveys and qualitative interviews). Such methods are susceptible to the potential effects of forgetting, distortion, and social desirability bias. Further, when imprecise, uncalibrated scales are used, self-report methods can be hampered by “arbitrary metrics”, in which responses are not grounded in the behaviors they are purported to measure [12]. Consequently, it is difficult to know how well the retrospective reports of physical therapists regarding their use of psychological techniques reflect their actual use of the techniques.

According to Annear et al. [13], the most widely used survey instrument for assessing the use of psychological techniques by physical therapists has been the Physiotherapists and Sport Psychology Questionnaire (PSPQ) [14], which was adapted from a comparable instrument for athletic trainers [15]. On the PSPQ, physical therapists indicate the extent to which they use 13 different techniques on a 5-point scale with response options of “never” (1), “25% of sessions” (2), “50% of sessions” (3), “75% of sessions” (4), and “100% of sessions” (5). In studies where the PSPQ has been administered, techniques such as goal setting, creating variety in rehabilitation exercises, and encouraging positive self-talk have been reported as being used, on average, in more than 75% of sessions [14,15,16,17,18,19]. In contrast, techniques such as teaching emotional control strategies [17,19], reducing depression, improving social support, mental rehearsal/visualization, and relaxation [19] have been reported as being used, on average, in fewer than 25% of sessions.

Because the response options on the PSPQ provide a precise estimate of the use of any given technique, the instrument does not involve arbitrary metrics. Because it is administered retrospectively across the entirety of the previous physical therapy sessions of respondents, however, the PSPQ is subject to the limitations of self-report measures. Given these limitations, the purpose of the current study was to examine the correspondence (i.e., strength of association) and concordance (i.e., discrepancy) of physical therapists’ responses on the PSPQ with their use of the same techniques as reported concurrently by the physical therapists and their patients. It was anticipated that the findings would provide an estimate of the extent to which PSPQ responses accurately reflect the psychological intervention content of physical therapy sessions.

## 2. Materials and Methods

### 2.1. Setting

This study was conducted in several private physical therapy clinics.

### 2.2. Design

A nested/hierarchical correlational research design was used in this study.

### 2.3. Participants

Participants were physical therapists (*n* = 14, 7 women and 7 men) affiliated with several private clinics and their patients (*n* = 306, 168 women and 138 men). The physical therapists reported ranges of 25–57 (*M* = 39.29, *SD* = 10.58) years of age and 1–32 (*M* = 14.07, *SD* = 9.84) years of experience in physical therapy. All but one physical therapist identified as non-Hispanic or Latino and all identified their race as White. Each physical therapist had an average of 21.86 (*SD* = 9.51, range = 9–33) patients enrolled in this study. A sample of 13 physical therapists was needed to obtain 80% power for correlations reflecting at least 50% shared variance (i.e., *r* = 0.71) between retrospective and concurrent reports of the use of psychological techniques by physical therapists.

The patients reported a range of 10–92 (*M* = 52.36, *SD* = 10.58) years of age. They indicated that they had spent from 1 to 104 (*M* = 6.25, *SD* = 9.84) weeks working with their physical therapist and that they were receiving physical therapy for conditions involving multiple body regions (*n* = 74), the knee (*n* = 73), shoulder (*n* = 57), back (*n* = 33), hip (*n* = 23), ankle (*n* = 17), foot (*n* = 13), neck (*n* = 9), calf (*n* = 1), elbow (*n* = 1), thigh (*n* = 1), or upper arm (*n* = 1). Nearly all patients identified as non-Hispanic or Latino (*n* = 295), with the remaining patients identifying as Hispanic or Latino (*n* = 5) or not specifying an ethnicity (*n* = 6). Nearly all patients identified as White (*n* = 290), with the remaining patients identifying as African American/Black (*n* = 6), Asian (*n* = 3), Native American (*n* = 1), other (*n* = 2), or unspecified (*n* = 4). In terms of sport participation, patients identified as competitive athletes (*n* = 31), recreational athletes (*n* = 109), nonathletes (*n* = 163), or unspecified (*n* = 3).

### 2.4. Instruments and Procedure

This study was approved by the Institutional Review Board at Springfield College. Informed consent was obtained from physical therapists and patients, and the rights of both groups of participants were protected. Data were collected from August 2019 through November 2019. Initially, a convenience sample of physical therapists was recruited. At the outset of this study, the physical therapists completed an informed consent document, a questionnaire requesting demographic information (i.e., age, gender, race/ethnicity, number of years of experience as a physical therapist) and the PSPQ [14]. Of the 13 PSPQ items assessing the physical therapists’ retrospectively reported use of psychological techniques in physical therapy sessions, an item pertaining to keeping patients involved with a team was not included in the current study because it was not relevant to the context in which this study was conducted, a practice consistent with that applied by Knuth et al. [18] for a sample that did not consist solely of athletes. Although the PSPQ has been widely used [13], no psychometric data are available for the instrument. 

Immediately after each physical therapy session during the study period (i.e., approximately 3 weeks at each clinic, which was long enough for the physical therapists to cycle through their caseload), the physical therapists completed a checklist with items adapted from the PSPQ. On the checklist, the physical therapists indicated which (if any) of the 12 psychological techniques listed on the PSPQ that they had used during the session. Thus, the checklist differed from the PSPQ only in terms of temporal perspective (i.e., concurrent versus retrospective). Sessions were approximately one hour in duration. Patients (and, for patients under 18 years of age, their parents/guardians) completed an informed consent document and a questionnaire requesting demographic information (e.g., age, gender, ethnicity, race, level of sport involvement, body region being treated, and duration of treatment with their current physical therapist) before a regularly scheduled physical therapy appointment. Immediately after the session, patients completed the same checklist that the physical therapists completed to indicate which of the 12 psychological techniques listed on the PSPQ that their physical therapist had applied during the session.

### 2.5. Data Analysis

Quantitative data were entered into the IBM Statistical Package for the Social Sciences (SPSS) version 24. Data were screened for accuracy and completeness. A series of independent-samples *t*-tests was conducted to compare patients who identified as nonathletes with those who identified as competitive or recreational athletes on the physical therapists’ use of psychological techniques during rehabilitation sessions as reported by both physical therapists and patients. For each psychological technique, Spearman rho correlations were calculated among physical therapists’ PSPQ responses (converted to the percentage of sessions they reported using the techniques), physical therapists’ self-reported use of the psychological technique across sessions, and patients’ reports of their respective physical therapists’ use of the psychological technique. The analysis was used to assess the correspondence between retrospective PSPQ responses and concurrent accounts of psychological techniques used during physical therapy sessions. Correspondence was also assessed more generally with a multilevel regression analysis in which the percentages of sessions in which physical therapists and patients reported using the 12 psychological techniques assessed on the PSPQ were used as predictors of the percentage of sessions that the physical therapists reported using the techniques on the PSPQ. Multilevel regression was suitable for this analysis due to the fact that the data are nested. Specifically, there were 12 psychological techniques nested within each of 14 physical therapists, for a total of 168 observations. Within each method of assessing physical therapists’ use of psychological techniques during physical therapy sessions (i.e., the PSPQ, the physical therapist checklist, and the patients’ checklist), Spearman correlations were calculated to examine the extent to which the techniques were reported as being implemented together.

Concordance among physical therapists’ PSPQ responses with respect to the percentage of sessions they reported using each technique and the physical therapists’ checklist (PTCL) and the patients’ checklist (PCL) responses regarding the physical therapists’ use of the techniques during actual sessions was examined with a series of repeated-measures analyses of variance (ANOVAs) and follow-up Bonferroni pairwise comparisons for significant *F*-values. Alpha level for determining statistical significance in all analyses was set at 0.05. In addition, Bland–Altman plots were created to visually examine pairwise concordance among physical therapists’ PSPQ responses, the physical therapists’ checklist responses, and the patients’ checklist responses aggregated across items.

## 3. Results

Statistical analyses were performed on a sample of 14 physical therapists and 306 patients. Complete data sets were obtained for all participants. No significant differences were obtained in the series of independent-samples *t*-tests comparing participants who identified as athletes with those who identified as nonathletes on the behavioral checklist items. Consequently, the data for both groups were considered together in subsequent analyses. As shown in Table 1, the correspondence between retrospective (PSPQ) and concurrent (checklist) measures of use of psychological techniques in orthopedic and sports physical therapy was generally weak. Correlations between PSPQ scores and PTCL scores (*M* = 0.31, *SD* = 0.25) were strong for encouraging positive self-talk, but moderate at best for the rest of the psychological techniques. Correlations between PSPQ scores and PCL scores (*M* = 0.03, *SD* = 0.26) were moderate for muscular relaxation techniques and weak at best for the remaining psychological techniques, with 5 of the 12 associations in the negative direction. Correlations between PTCL and PCL scores (*M* = 0.42, *SD* = 0.12) were generally moderate, with none of the values exceeding 0.58.

In the more general assessment of the correspondence between retrospective and concurrent measures of use of psychological techniques, the multilevel regression model with the physical therapists’ checklist and the patients’ checklist scores as predictors of PSPQ scores (i.e., with all 12 psychological techniques nested within each of the physical therapist participants) showed that the physical therapists’ checklist scores were significant predictors of PSPQ scores, *b* = 0.42, *SE* = 0.11), *t*(152) = 4.00, *p* = 0.0001. The patients’ checklist scores were not significant predictors of PSPQ scores, *b* = 0.23, *SE* = 0.14, *t*(152) = 1.71, *p* = 0.09.

Results of the analyses in which Spearman correlations were calculated within each method of assessing physical therapists’ use of psychological techniques during physical therapy sessions (i.e., the PSPQ, the physical therapist checklist, and the patients’ checklist) are presented in Table 2, Table 3 and Table 4, respectively. Fewer than one-quarter of the correlations were statistically significant, and only two of the significant correlations were negative.

In the concordance analyses, significant effects were found in the repeated-measures ANOVAs for 7 of the 12 psychological techniques (i.e., variety in exercises, encouraging positive self-talk, teaching emotional control strategies, muscular relaxation techniques, visualization, improving social support, and reducing depression). As shown in Table 5, physical therapists’ PSPQ scores were significantly higher than their checklist scores for five psychological techniques (i.e., encouraging positive self-talk, teaching emotional control strategies, visualization, improving social support, and reducing depression) and significantly lower than their checklist scores for one psychological technique (i.e., variety in exercises). On average, physical therapists’ PSPQ scores were 13 (*SD* = 13, range = −15 to 35) percent higher than their checklist scores. The physical therapists’ checklist scores differed significantly from the patients’ checklist scores for two psychological techniques (i.e., teaching emotional control strategies and muscular relaxation techniques) and, on average, were 6 (*SD* = 10, range = −24 to 14) percent lower than the patients’ checklist scores.

**Figure 1:**
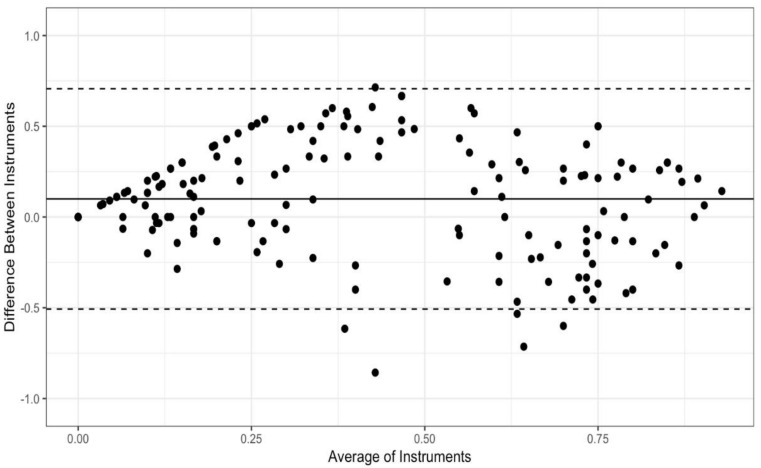
Bland-Altman plot for the PCL and the PTCL. Solid horizontal line represents the mean difference between instruments (the PCL minus the PTCL); dashed lines represent the 95% CI of differences.

**Figure 2:**
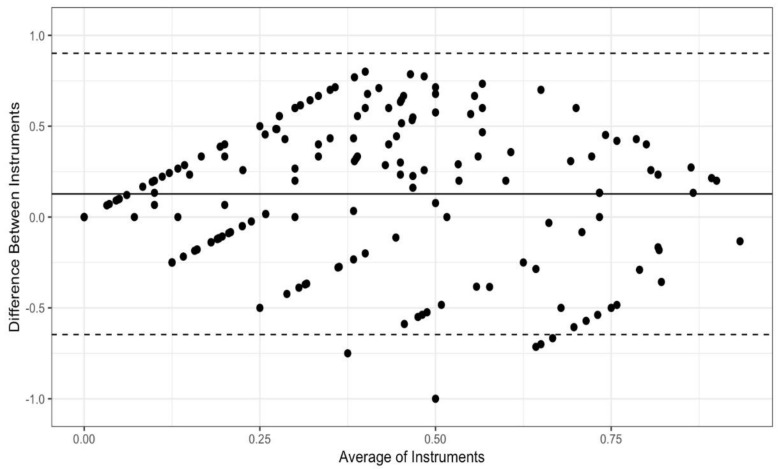
Bland-Altman plot for the PCL and the PSPQ. Solid horizontal line represents the mean difference between instruments (the PCL minus the PSPQ); dashed lines represent the 95% CI of differences.

**Figure 3:**
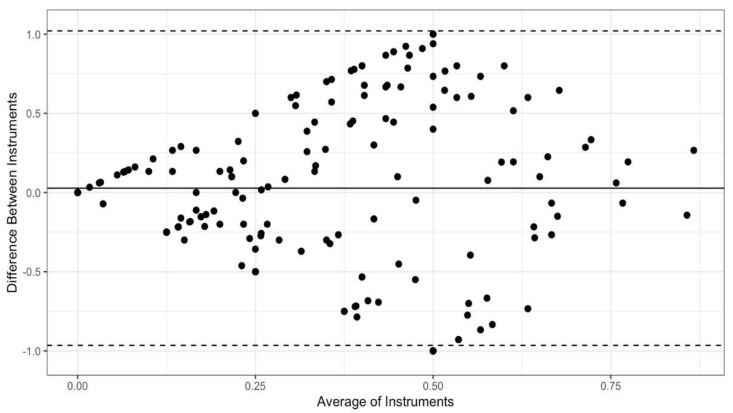
Bland-Altman plot for the PTCL and the PSPQ. Solid horizontal line represents the mean difference between instruments (the PTCL minus the PSPQ); dashed lines represent the 95% CI of differences.

Bland-Altman plots were created to visually examine concordance between each pair of instruments: the PCL and the PTCL (Figure 1), the PCL and the PSPQ (Figure 2), the PTCL and the PSPQ (Figure 3). These plots include all 14 participants and 12 variables combined. The mean difference (solid horizontal line) can be interpreted as bias toward one instrument relative to the other. Figure 1 and Figure 2 show that PCL scores tended to be higher than PTCL and PSQP scores by means of 0.10 and 0.13, respectively. By comparison, Figure 3 shows only a slight bias toward the PTCL, relative to the PSPQ (*M* = 0.03). Large variability in difference scores is an indicator of poor concordance. It is apparent from the confidence intervals shown in Figure 1, Figure 2 and Figure 3 that the variability is the least for the PCL and the PTCL (*SD* = 0.31; 95% CI [−0.51, 0.71]; Figure 1). By comparison, the variability in differences is particularly high, suggesting poor concordance, for the PCL and the PSPQ (*SD* = 0.39; 95% CI [−0.65, 0.90]; Figure 2) and the PTCL and the PSPQ (*SD* = 0.51; 95% CI [−0.96, 1.02]; Figure 3). The latter confidence interval suggests that it would not be uncommon for PTCL and PSPQ scores to differ by the maximum possible value of 1 (or 100%). As shown in Figure 3, there were many cases in which the instruments differed by 75% or more. 

## 4. Discussion

The primary aim of the current study was to assess the correspondence and concordance of physical therapists’ retrospective responses to PSPQ items with concurrent responses to the same items by physical therapists and their patients. From a purely descriptive standpoint, the findings for the PSPQ in the current study are highly consistent with those obtained in previous investigations. Parallel to the results of previous research, goal setting, positive self-talk, effective communication, and variety in rehabilitation exercises were reported as commonly used [13,14,16,17,18,19] and teaching emotional control strategies and reducing depression were reported as infrequently used [13,14,16,17,18,19] by the physical therapists. A different perspective emerged, however, when the current PSPQ data were examined alongside the checklist data and the correspondence and concordance between physical therapists’ retrospectively reported use of psychological techniques during rehabilitation sessions (on the PSPQ) and their concurrently reported use of the techniques as indicated by themselves and their patients were assessed. Although associations between PSPQ scores and physical therapists’ concurrent reports of psychological technique use were generally positive and of moderate strength, PSPQ scores were essentially unrelated to patients’ concurrent reports of the same techniques. With respect to concordance, significant discrepancies between PSPQ scores and physical therapists’ self-reported use of psychological techniques during rehabilitation sessions were found for most of the techniques assessed. 

It is possible that the general lack of correspondence and concordance between physical therapists’ retrospective and concurrent reports resulted from the physical therapy sessions in this study not being representative of the previous sessions that served as the foundation for PSPQ scores. It is also possible, however, that characteristics of the PSPQ may have contributed to the modest associations and significant discrepancies between retrospective and concurrent reports of the use of psychological techniques. Specifically, the retrospective nature of the PSPQ may have led to the trend toward physical therapist “overestimation” of their use of psychological techniques on the PSPQ relative to the (concurrent) checklist. Further, because definitions of the psychological techniques are not provided on the PSPQ (and, by extension, the checklist), physical therapists may respond inconsistently to the items over time and across patients and sessions. Even without the retrospection inherent in the PSPQ, the average correlation between the physical therapists’ and the patients’ checklist scores (i.e., 0.42) was remarkably low given that the physical therapists and patients were reporting on the same sessions that had concluded only moments earlier. 

In the absence of definitions of the psychological techniques, some of the PSPQ items are especially unclear. For example, it is unclear whether “encouraging positive self-talk” is interpreted as “encouraging patients to maintain a positive attitude” or “encouraging patients explicitly to say things to themselves that are positive.” Similarly, it is unclear whether “improving social support” is interpreted as “being supportive of patients” or “doing something to increase the quantity and/or quality of social support patients receive from others.” The extent to which the items “teaching emotional control strategies,” reducing stress and anxiety,” and “reducing depression” are interpreted as involving the initiation of behaviors with the primary intent of altering the emotional control, stress/anxiety, and depression, respectively, of patients (versus simply trying to cheer patients up or help them feel temporarily less distressed) is also unclear. It is worth noting that even some of the more seemingly clear and straightforward PSPQ items (e.g., “variety in exercises”, “use of goals”, “muscular relaxation strategies”, and “visualization”) displayed weak correspondence and/or poor concordance with the physical therapists’ checklist responses.

In light of the current findings, it is reasonable to question whether the PSPQ (and similar instruments involving retrospective self-report) provides an accurate assessment of the psychological techniques used by physical therapists. The accuracy and sensitivity of the PSPQ could conceivably be improved by furnishing definitions (and possibly examples) of the techniques that constitute the questionnaire items in concrete behavioral terms. Before relying exclusively on the PSPQ to assess physical therapists’ use of psychological techniques, however, it is necessary to validate the questionnaire (and corresponding checklist) against objectively assessed use of psychological techniques by physical therapists. Direct observation of psychological technique usage by physical therapists—either live or via audio or video recording [20]—during rehabilitation sessions could be implemented as a standard against which PSPQ (and checklist) responses could be calibrated. Chart reviews might also prove helpful in this regard, but it is possible that some or even most of the psychological techniques may not be routinely recorded by physical therapists. Only after PSPQ scores demonstrate strong correspondence and good concordance with objective indicators of the use of psychological techniques in physical therapy sessions can the PSPQ be regarded as an accurate measure of its intended construct.

It is important to acknowledge several limitations of the current study. First, although the size of patient sample was relatively large, the size of the physical therapist sample was relatively small and obtained through convenience sampling. Larger, more representative samples of physical therapists should be used in future research to enhance both statistical power and generalizability of the results. Second, only a single session was monitored for each patient. Subsequent investigations can be strengthened by examining the use of psychological techniques by physical therapists in multiple sessions for each patient, thereby expanding the assessment window and enabling conclusions based on a larger and potentially more reliable behavioral sample. Third, in lacking concrete behavioral definitions, the checklists developed for the current study suffered from the same limitations in item clarity as the questionnaire on which they were based. If the checklist approach to concurrent assessment of the use of psychological techniques is used in future research, definitions of the techniques should be provided. Adding such definitions could help make the assessment more sensitive to the construct being measured.

## 5. Conclusions

The current findings suggest that the most common method of assessing the use of psychological techniques by physical therapists—namely retrospective self-report with surveys such as the PSPQ—does not yield accurate results. Although retrospective surveys are cost- and time-effective, minor modifications and extensive validation research are needed before they can be used with confidence for their intended purpose. More refined measurement of psychological technique use by physical therapists can inform educational efforts for physical therapy students and professionals [21,22,23,24,25] and facilitate discussion about which psychological techniques are most appropriate for use by physical therapists and under which circumstances [26]. Such developments may ultimately help physical therapists to better serve the patients with whom they work.

## Figures and Tables

**Table 1:** Spearman correlations between physical therapists’ responses to the PSPQ items and physical therapists’ and patients’ responses to the behavioral checklist items.

Variable	r(PSPQ-PTCL)	r(PSPQ-PCL)	r(PTCL-PCL)
Variety in exercises	0.23	0.13	0.4
Use of goals	0.09	−0.2	0.12
Encouraging positive self-talk	0.74 ^†^	0.25	0.57 *
Encouraging communication	0.51	−0.3	0.34
Enhancing self-confidence	0.32	0.17	0.49
Teaching emotional control strategies	0.12	−0.25	0.42
Reducing stress and anxiety	−0.27	0.05	0.57 *
Muscular relaxation techniques	0.12	0.49	0.53
Relaxation techniques	0.51	0.35	0.34
Visualization	0.24	−0.35	0.35
Improving social support	0.38	−0.04	0.44
Reducing depression	0.44	0.08	0.48

Note. PSPQ = Physiotherapists and Sport Psychology Questionnaire; PTCL = Physical Therapist Behavioral Checklist; PCL = Patient Behavioral Checklist. * *p* < 0.05, ^†^ *p* < 0.005.

**Table 2:** Spearman correlations among responses to Physiotherapists and Sport Psychology Questionnaire items.

Item	1	2	3	4	5	6	7	8	9	10	11
Variety in exercises											
2. Use of goals	0.55										
3. Encouraging positive self-talk	0.65	0.51									
4. Encouraging communication	−0.11	0.11	0.00								
5. Enhancing self-confidence	−0.21	−0.31	−0.02	0.49							
6. Teaching emotional control strategies	0.10	0.18	−0.33	−0.18	−0.08						
7. Reducing stress and anxiety	0.06	−0.26	−0.29	−0.01	0.42	0.38					
8. Muscular relaxation Techniques	0.69	0.76	0.53	0.40	0.02	0.20	−0.02				
9. Relaxation techniques	0.34	0.33	0.06	−0.05	−0.05	0.78	0.39	0.40			
10. Visualization	0.22	0.32	0.00	−0.22	0.21	0.75	0.61	0.32	0.73		
11. Improving social support	0.19	0.14	−0.10	0.19	0.39	0.49	0.87	0.32	0.51	0.71	
12. Reducing depression	0.40	0.24	0.13	0.42	0.27	−0.09	0.39	0.62	−0.04	0.11	0.55

Note. *N* = 14; correlations > |0.54| are significant at *p* < 0.05; correlations > |0.68| are significant at *p* < 0.01.

**Table 3:** Spearman correlations among responses to Physical Therapist Behavioral Checklist items.

Item	1	2	3	4	5	6	7	8	9	10	11
Variety in exercises											
2. Use of goals	0.38										
3. Encouraging positive self-talk	−0.02	−0.06									
4. Encouraging communication	−0.02	−0.13	0.63								
5. Enhancing self-confidence	−0.20	−0.29	0.75	0.80							
6. Teaching emotional control strategies	0.31	0.43	0.20	0.04	−0.01						
7. Reducing stress and anxiety	0.03	−0.03	0.67	0.41	0.41	0.01					
8. Muscular relaxation Techniques	−0.25	−0.13	0.31	0.19	0.16	0.09	0.48				
9. Relaxation techniques	−0.05	0.23	−0.05	−0.19	−0.35	0.26	0.34	0.79			
10. Visualization	0.10	0.26	0.30	−0.09	0.02	0.81	0.18	0.24	0.37		
11. Improving social support	0.63	−0.01	−0.11	0.03	−0.14	0.23	0.29	0.17	0.25	0.21	
12. Reducing depression	0.34	0.12	0.15	0.13	−0.04	0.12	0.65	0.03	0.21	0.26	0.52

Note. *N* = 14; correlations > |0.54| are significant at *p* < 0.05; correlations > |0.68| are significant at *p* < 0.01.

**Table 4:** Spearman correlations among responses to Patient Behavioral Checklist items.

Item	1	2	3	4	5	6	7	8	9	10	11
Variety in exercises											
2. Use of goals	−0.08										
3. Encouraging positive self-talk	0.03	0.00									
4. Encouraging communication	0.08	−0.20	0.62								
5. Enhancing self-confidence	−0.20	0.19	0.79	0.66							
6. Teaching emotional control strategies	−0.21	0.04	0.23	0.22	0.67						
7. Reducing stress and anxiety	−0.30	−0.19	0.53	0.73	0.67	0.51					
8. Muscular relaxation Techniques	−0.69	0.06	−0.13	0.19	0.09	0.01	0.35				
9. Relaxation techniques	−0.20	−0.56	0.06	0.57	0.13	0.07	0.55	0.59			
10. Visualization	0.02	0.13	0.42	0.28	0.67	0.57	0.31	−0.17	0.07		
11. Improving social support	−0.02	−0.17	0.26	0.33	0.56	0.79	0.58	−0.15	0.14	0.67	
12. Reducing depression	0.11	−0.25	0.61	0.57	0.79	0.72	0.54	−0.19	0.29	0.80	0.74

Note. *N* = 14; correlations > |0.54| are significant at *p* < 0.05; correlations > |0.68| are significant at *p* < 0.01.

**Table 5:** Means and standard deviations for physical therapists’ responses to the PSPQ items, physical therapists’ responses to behavioral checklist items, and patients’ responses to the behavioral checklist items.

Item	*M*PSPQ	*SD*PSPQ	*M*PTCL	*SD*PTCL	*M*PCL	*SD*PCL	*ES*
Variety in exercises	0.82 *	0.18	0.97 ^†^	0.06	0.96 ^†^	0.05	0.28
Use of goals	0.79 *	0.22	0.84 *	0.18	0.70 *	0.13	0.09
Encouraging positive self-talk	0.86 *	0.19	0.66 ^†^	0.3	0.61 ^†^	0.15	0.21
Encouraging communication	0.80 *	0.17	0.72 *	0.26	0.77 *	0.1	0.03
Enhancing self-confidence	0.73 *	0.23	0.58 *	0.29	0.61 *	0.16	0.08
Teaching emotional control strategies	0.38 *	0.21	0.03 ^‡^	0.06	0.11 ^†^	0.07	0.56
Reducing stress and anxiety	0.45 *	0.3	0.32 *	0.24	0.56 *	0.14	0.16
Muscular relaxation Techniques	0.41 *^,†^	0.27	0.30 ^†^	0.26	0.50 *	0.15	0.12
Relaxation techniques	0.34 *	0.25	0.22 *	0.28	0.40 *	0.12	0.10
Visualization	0.29 *	0.22	0.12 ^†^	0.17	0.14 *^,†^	0.08	0.18
Improving social support	0.30 *	0.28	0.07 ^†^	0.15	0.12 *^,†^	0.1	0.23
Reducing depression	0.29 *	0.26	0.06 ^†^	0.12	0.13 *^,†^	0.09	0.25

Note. PSPQ = Physiotherapists and Sport Psychology Questionnaire; PTCL = Physical Therapist Behavioral Checklist; PCL = Patient Behavioral Checklist; means with different superscripts differ significantly in pairwise comparison (*p* < 0.05); *ES* = eta-squared effect size.

## Data Availability

The data presented in this study are available on request from the corresponding author.
